# When African and European Lineages Meet: The Genetic Landscape of Honey Bees in Argentina

**DOI:** 10.1002/ece3.72233

**Published:** 2025-09-28

**Authors:** Arian Avalos, Alejandra Scannapieco, A. Carolina Monmany‐Garzia, Ravi Kiran Donthu, José Marcelino, Rosanna Giordano, Tugrul Giray, Alberto Galindo‐Cardona

**Affiliations:** ^1^ Honey Bee Breeding Genetics and Physiology Unit USDA‐ARS Baton Rouge Louisiana USA; ^2^ Instituto de Genética “E. A Favret”, GV AL IABIMO, INTA – CONICET Buenos Aires Argentina; ^3^ Instituto de Ecología Regional IER – CONICET Tucumán Argentina; ^4^ Centre for Life Sciences Mahindra University Hyderabad India; ^5^ Division of Plant Industry Florida Department of Agriculture and Consumer Services Gainesville Florida USA; ^6^ Puerto Rico Science, Technology and Research Trust and Know Your Bee, Inc. San Juan Puerto Rico USA; ^7^ Departamento de Biología Universidad de Puerto Rico San Juan Puerto Rico USA

**Keywords:** ecoregion, genetic diversity, honeybee, marker panel, population genetics

## Abstract

Argentina has a complex and diverse landscape of honeybee ( *Apis mellifera* sp.) populations shaped by historic introductions and hybridization between Africanized (AHB) and European (EHB) lineages. While a latitudinal cline of Africanization has been documented, the adaptive consequences of this genetic admixture and its implications for local beekeeping practices remain poorly understood. In this study, we provide a more in‐depth analysis of Argentine honeybee populations using recently published data from a panel of 272 SNP markers across five ecoregions to: (1) quantify how ancestry proportion (African A, European C/M) varies along geographic gradients, (2) assess whether ecoregion boundaries influence population structure, and (3) evaluate the potential trade‐offs between AHB and EHB ancestry in hybrid genomes. Our results confirm a strong latitudinal pattern of Africanization but reveal novel complexity, with C‐lineage ancestry inversely correlated with A‐lineage contributions while M‐lineage ancestry remains independent. We also detected trace contributions from the O lineage (Middle Eastern), highlighting Argentina's complex admixture history. Despite Argentina's diverse ecoregions, we find limited evidence for ecotype‐specific differentiation, suggesting gene flow may outweigh local adaptation—though sampling limitations warrant caution. By linking genetic patterns to apicultural relevance (e.g., AHB's northward expansion and hybridization zones), this study provides a framework for conserving genetic diversity and managing hive productivity across environmental gradients. Further genome‐wide analyses are needed to disentangle adaptive traits in this understudied yet economically critical pollinator system.

## Graphical abstract

Using a 272‐SNP panel, we explored how Africanized (AHB) and European (EHB) honeybee populations interact across Argentina's diverse landscapes. A clear latitudinal pattern in Africanization, with an interesting inverse relationship between African (A) and European (C) ancestry. Small but consistent contributions from Middle Eastern (O) lineage genes and surprisingly little genetic differentiation between ecoregions.

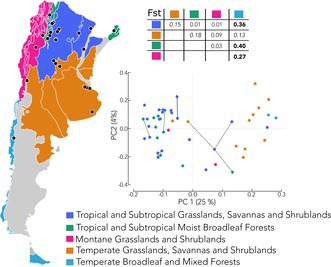

## 1 Introduction

Honeybees ( *Apis mellifera* sp.) are a diverse group, comprising over 24 subspecies native to regions across Europe, the Middle East, and Africa. Genetic analyses of the species have revealed up to seven distinct genetic subgroups (A, C, M, O, Y, U, L) largely defined by both mitochondrial lineages and similarities in markers across the nuclear genome, both of which coincide with major ecological expansions of the original species range (Ruttner et al. 1978; Ruttner 1988; Whitfield et al. 2006; Dogantzis et al. 2021). In contrast, the most recent major honeybee expansion has been facilitated by human intervention, where several populations have been introduced to the Western Hemisphere over the course of 400 years of human colonialism and subsequent immigration. During this time, small populations of many of these subspecies were intentionally brought over to the Western Hemisphere, resulting in highly admixed continental populations with various degrees of genetic ancestries contributing to their genetic variation (Whitfield et al. 2006; Wallberg et al. 2014).

Presently, it is known that largely four (A, C, M, O) of the seven genetic subgroups are represented in populations of the Western Hemisphere (Morse et al. 1973; Ruttner et al. 1978; Ruttner 1988; Sheppard 1989; Whitfield et al. 2006; Carpenter and Harpur 2021; Dogantzis et al. 2021). The distribution of ancestral genetic composition in these populations is known to vary with ecological and historical factors, with the first confirmed European honeybees (EHB) introduction to North America from England around 1621 (Carpenter and Harpur 2021), though earlier introductions have been identified (De Jaime Lorén 2003), and subsequent introductions from Europe occurring in Mexico by 1830 (Bierzychudek 2011). Specifically, in Argentina, German migrants practicing beekeeping in Rio Grande do Sul (Brazil) since 1824 likely facilitated the spread of the honeybee into the country, and the introduction of various additional EHB subspecies, including *A. m. mellifera* (M group) from France in 1834 and *A. m. ligustica* (C group) from Italy in 1855 (Bierzychudek 1979; Requier 2019). Other EHB subspecies like *A. m. carnica* (C group), *A. m. caucasica* (O group), and *A. m. iberiensis* (M group) were introduced through crossbreeding efforts (Requier 2019). The late 1950s saw the introduction of *A. m. scutellata* Lepeletier, an African (A group) subspecies which readily mated with existing admixed populations, which resulted in the hybrid Africanized Honeybees (AHB) (Requier 2019).

The history of honeybee introductions to the Western Hemisphere is key to the understanding of honeybee populations that are present in Argentina, where their genetic history intertwines with a diverse range of ecoregions and a clear hybrid zone between EHB and AHB remains over the years (Whitfield et al. 2006; Calfee et al. 2020). Initial genetic studies of this hybrid zone revealed the coexistence of both EHB and AHB lineages, with a latitudinal cline from north to south indicating varying degrees of admixture (Sheppard et al. 1991; Whitfield et al. 2006). Recent studies have confirmed the presence of a latitudinal boundary for Africanization, clearly showing that AHB are found in to the north of Argentina and EHB in the south, with a zone of hybridization between 28 and 35 degrees south (Agra et al. 2018; Calfee et al. 2020; Porrini et al. 2022). This has been supported by ensuing updates on Africanization and in one analysis of the province of Buenos Aires, aids in understanding the southward movement of AHB (Genchi García et al. 2018), which is crucial for comprehending the dynamics of this hybrid zone. More recently, genome‐wide analyses show that at the population genetics level, distribution is a smooth but stable transition likely limited by environmental factors (Calfee et al. 2020). Given this history of introductions, Argentina beekeepers have extensive experience managing admixed *A. mellifera* populations (Dietz et al. 1985). However, critical gaps persist in understanding how AHB are distributed across the country and how this spatial patterning influences both ecological adaptations and apicultural practices. Specifically, the interaction between AHB hybridization gradients and ecoregion‐specific selective pressures—such as climate, floral resources, or disease prevalence—remains unresolved (Calfee et al. 2020). In the present study, we capitalize on a recently published 272 single nucleotide polymorphism (SNP) panel (Donthu et al. 2024) optimized to trace Africanization to examine populations across Argentina. We address three key questions: (1) How do ancestry proportions (A, C, M, and O lineages) vary across Argentina's latitudinal and ecoregional gradients? (2) Does ecoregion boundary predict genetic differentiation, or is gene flow homogenizing populations? (3) What are the practical implications of these for beekeeping strategies and conservation? By integrating high‐resolution genetic data with Argentina's ecographic diversity, this study advances the characterization of honeybee population structure while providing actionable insights for sustainable management of hybridized colonies.

## 2 Materials and Methods

### 2.1 Data Collection and Processing

**FIGURE 1:**
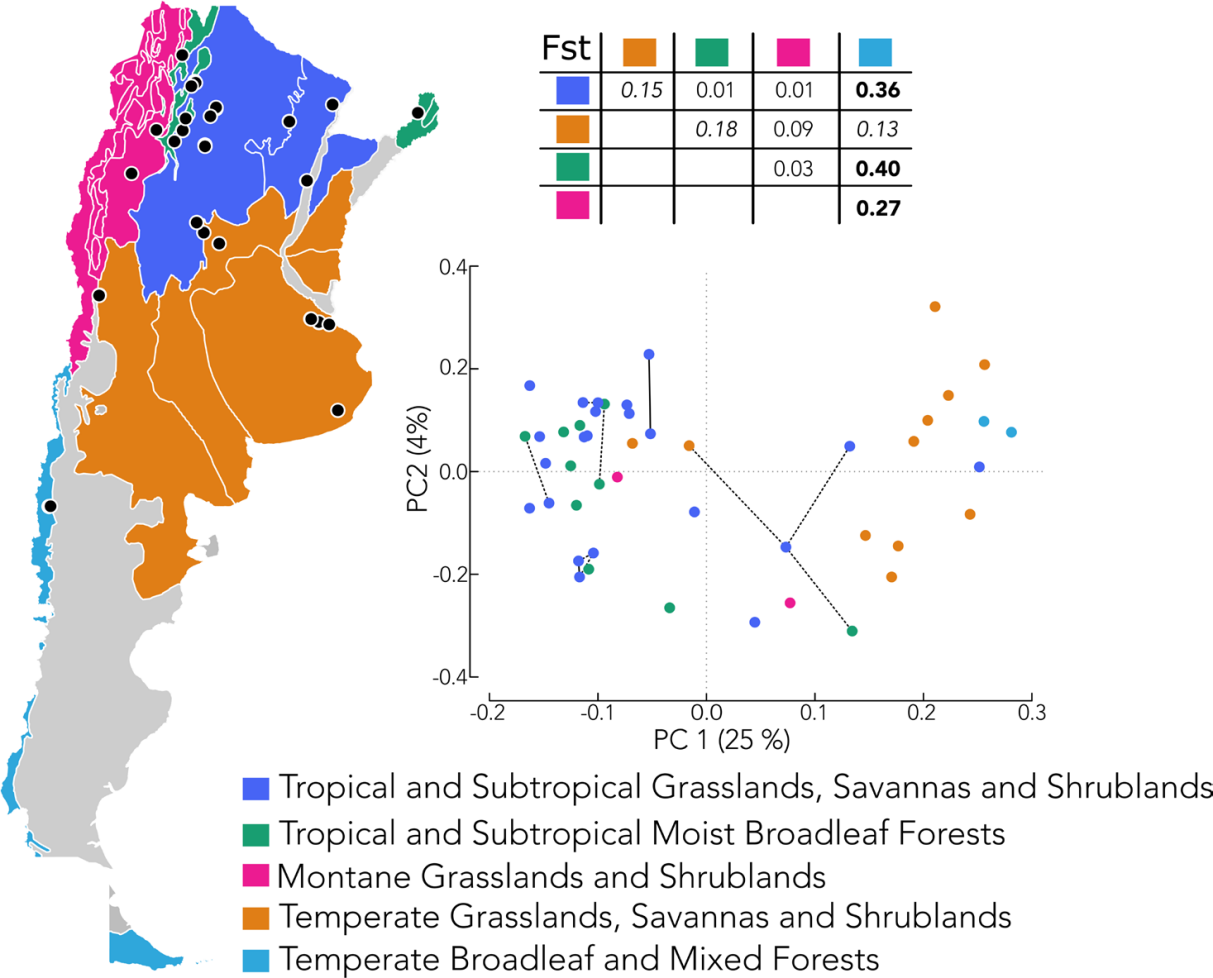
Geographic and genetic information for Argentina sample set. The figure illustrates geographic locations where each sample was collected in Argentina. The map show is sectioned by ecoregions. A total of seven ecoregions were identified across Argentina, though in this study samples from only five were collected (shown in color). The figure also highlights genetic similarity via PCA of samples with each point in the plot corresponding to a sample and all samples colored by ecoregion. Lines in the PCA correspond to levels of kinship with solid black lines identifying first‐degree kinship while dotted black lines identify second‐degree kinship. In addition, an Fst table for the genetic differentiation between ecoregions is provided.

A total of 47 honeybee worker pupae samples (one per colony) were collected in Argentina from 13 provinces and five ecoregions of the seven generally described for the country (see Figure 1). We limited our analysis to one individual per colony to prevent oversampling of maternal alleles. Genomic DNA from half the thorax of an individual honeybee worker pupa was extracted using the DNeasy extraction kit from QIAGEN with the animal tissue protocol. Resulting DNA quality and concentration were examined using agarose gel electrophoresis (1%), NanoDrop (NanoDrop ND‐1000), and Qubit Fluorometer (Invitrogen), following the manufacturer's instructions.

### 2.2 Genotyping

Genotyping for the Argentina sample set was conducted as described in Donthu et al. (2024). In brief, the 272 SNP set was selected using a combination of prior studies and focusing on those that were most informative in differentiating local honeybee populations. DNA from the Argentina samples was ultimately genotyped as part of this larger set of 874 samples from across the world using primers developed and deployed as part of a Fluidigm genotyping system (details in Donthu et al. 2024).

### 2.3 Genetic Diversity

To examine genetic diversity, we first conducted a Principal Component Analysis (PCA) of the samples × SNPs dosage matrix from Donthu et al. (2024). This was achieved through functions available as part of the *SNPRelate* package in R (Zheng et al. 2012), allowing us to visualize the degree of genetic similarity across samples. For the analysis of specific associations, we also calculated kinship estimates via the KING Method of Moments approach (Manichaikul et al. 2010) using the snpgdsIBDKING() function of the *SNPRelate* package to identify samples with putative filial ties.

### 2.4 Ancestry

The ancestral contribution in our data set was examined firstly by developing a reference panel from the broader, worldwide genotypes in Donthu et al. (2024). Specifically, we identified and selected a set of representing samples from ancestral honeybee ranges that are unlikely to be affected by introgression. Our first selection criteria to establish this set focused on country of origin due to the close ties between subspecies and geographic distribution. We then applied a second selection criterion that used the cluster classifications in Donthu et al. (2024). In this way, reference samples were confirmed by both geographic origin and genetic aggregation as per the HBeeID determination. Following curation, we further reduced the HBeeID assignations to broad genomic groupings (e.g., A, M, O, C as per Whitfield et al. 2006; Wallberg et al. 2014; Dogantzis et al. 2021) to broaden our reference groups.

**FIGURE 2:**
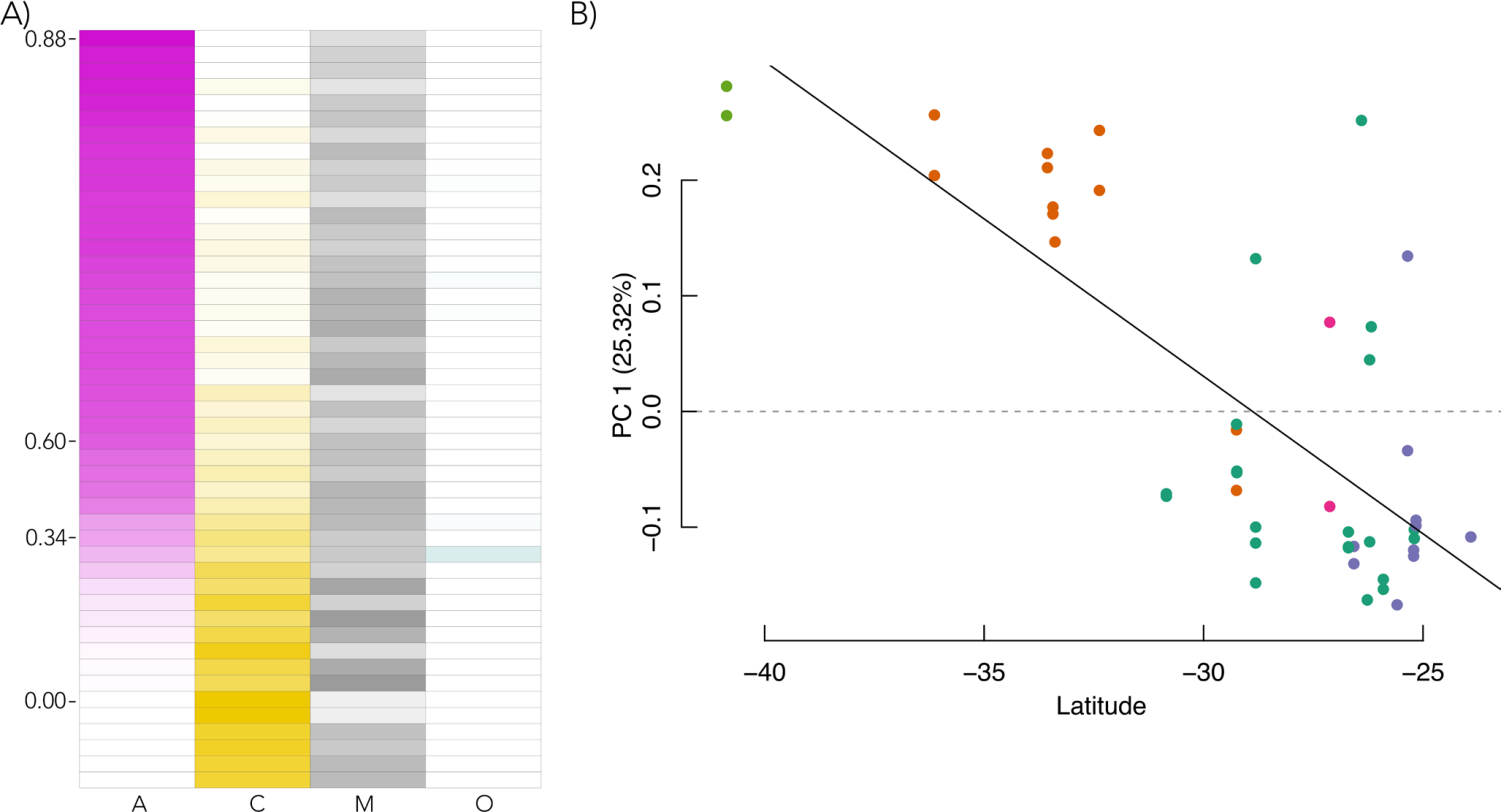
Proportional Ancestry and Broad Geographic Correlations. The figure illustrates two broad level analyses of genetic distribution. Panel A presents a matrix with rows corresponding to the 47 individuals in the Argentina sample set and columns corresponding to the proportion of allelic similarity to each reference ancestral group. Overall, we highlight that all three proportional ancestries previously described (A, C, M, O) are present, with some trade‐off observed between the proportion of A and C ancestry contribution. Panel B highlights the correlation between first principal component and Latitude; this relationship is also observed when applied to proportional ancestry.

The final reference sample set was processed following the methodology in Funkhouser et al. (2017) and Chinchilla‐Vargas et al. (2021). In brief, this approach runs a supervised linear analysis of genetic composition, which regresses per‐sample dosage to the allele frequencies of the reference set. This results in per‐sample proportions for each reference group. In this way, for our Argentine sample set, we can determine the proportion of each ancestry in each sample (Figure 2A).

### 2.5 Geography

We characterized the relationship between broad geography and genetic diversity by firstly correlating the principal components from the PCA to the corresponding latitude and longitude values for each sample. Two iterative correlational sweeps were conducted, one with latitude values and another with longitude values. Resulting *p*‐values were compared against a Bonferroni corrected alpha value of 0.001 (Figure 2B). Statistical differences between ecoregions were analyzed via F_ST_ analysis. Samples were sorted by ecoregion, and a pairwise analysis of F_ST_ was applied using the snpgdsFst() function in the *SNPRelate* package with the “W&H02” method parameter which uses the F_ST_ calculation in Weir and Hill (2002).

## 3 Results

We found no specific genetic grouping by ecoregion in the samples, though allelic divergence (Fst) was observed between some of the sampled ecoregions (Figure 1—table). In an analysis of kinship, we observed first‐degree kinship associations within ecoregions while second‐degree kinship associations were frequently found across ecoregions (Figure 1). Further analysis of broad geography showed only a significant correlation between the first principal component and latitudinal coordinate (Adjusted *R* ^2^ = 0.57, *p*‐value = 1.11E^−9^). No similar result was found with any of the other principal components and latitude or longitude coordinate values (Figure 1).

The analysis of ancestry showed two distinct findings. First, the proportion of A group ancestry also significantly correlates with only Latitude (Adjusted *R* ^2^ = 0.51, *p*‐value = 1.19E^−8^). Second, for this SNP set, there seems to be a trade‐off in the proportional representation between only A and C groups (Figure 2A), with the proportional contribution of the M group largely independent of the other backgrounds.

## 4 Discussion

The complex history of honeybee introductions in Argentina has created a unique genetic landscape where Africanized (AHB) and European (EHB) lineages coexist in a dynamic hybrid zone. Our findings confirm the well‐documented latitudinal cline of Africanization (Whitfield et al. 2006; Calfee et al. 2020; Zárate et al. 2022). We also demonstrate a strong inverse relationship between African (A) and European (C) ancestry across Argentine populations (Figure 2A), a pattern previously described in this population and others (Whitfield et al. 2006; Calfee et al. 2020; Zárate et al. 2022), which may suggest that either selective pressures or genome‐level incompatibilities (suggested in Gibson et al. 2015) may mediate ancestral contributions. In contrast, the M lineage shows consistent representation independent of A/C, potentially indicating neutral introgression or environment‐specific adaptive advantages (Figure 2A). Like Donthu et al. (2024), we detect O group contribution in the Argentina honeybee population, and our approach shows this is not regionally localized, but rather a small (< 5% ancestry proportion) but consistent contribution that could reflect ancestral admixture occurring prior to or during Argentina's colonial period. This was surprising, as past analyses of the Argentinian honeybee population did not identify O group mitotypes (Porrini et al. 2022), and nuclear analyses have not similarly detected it (Whitfield et al. 2006; Calfee et al. 2020). The persistence of O‐lineage alleles also raises questions about their functional role—whether they are neutral relics or contribute to local adaptation, such as arid‐climate resilience, a trait associated with O‐lineage bees in their native range (Ruttner 1988) (Figure 2A).

Argentina's dramatic tropical‐to‐temperate climate gradient makes this system a compelling example of how invasive organisms adapt across environmental clines. The spatial distribution of these genetic patterns—from AHB‐dominated northern populations to EHB‐dominated southern colonies—has direct implications for beekeeping challenges and conservation strategies, particularly as climate change alters selective pressures across these regions. Critically, the persistence of AHB ancestry in northern populations, despite decades of hybridization, underscores the adaptive potential of African‐derived traits (e.g., disease resistance, thermotolerance) and their relevance for beekeepers managing defensive colonies.

Despite Argentina's diverse ecoregions (Figure 1), we detected a minimal population structure tied to ecological boundaries. While allelic divergence was faintly detectable, small sample sizes in key regions (e.g., Temperate Broadleaf Forests) and persistent kinship networks across habitats suggest that gene flow—whether natural or transhumance—may homogenize populations faster than local adaptation can arise (Figure 1). However, the subtle divergence observed in better‐sampled ecoregions (e.g., Montane Grasslands) hints that environmental pressures could eventually drive differentiation, particularly if climate change alters floral resources or disease prevalence. The prospect of environmental factors shaping population boundaries within a contiguous geographic span is in concordance with past work (see Calfee et al. 2020). In their findings, Calfee and colleagues precisely highlight how ecological factors have effectively established the distribution bounds even for highly admixed populations undergoing rapid expansion. Our findings agree and indeed highlight that given enough time, these same ecological boundaries may further sub‐segment the existing hybrid population.

The dominance of AHB ancestry in northern Argentina poses both challenges and opportunities for apiculture. While Africanized bees are often more defensive, their resilience to parasites like *Varroa* mites (Genchi García et al. 2018) could benefit sustainable management. Conversely, southern populations with higher EHB ancestry may require targeted conservation to maintain genetic diversity, especially as AHB ranges expand southward. Our results highlight the need for region‐specific management strategies, such as breeding programs to temper defensiveness in AHB‐dominant areas or protecting EHB refugia in temperate zones.

To resolve the functional significance of the A‐C ancestry trade‐off, future work should integrate: (1) *genome‐wide scans* to identify loci under selection, (2) *phenotypic data* linking ancestry to traits like behavior or disease resistance, and (3) *longitudinal monitoring* of hybrid zones to track shifts in Africanization under climate change. By bridging genetic patterns with practical apiculture, this study lays the groundwork for a more nuanced understanding of honeybee evolution in Argentina—one that balances conservation priorities with the needs of a critical agricultural sector. Finally, the enigmatic O‐lineage ancestry, though minor, warrants targeted studies—especially to test whether its haplotypes may be adaptive across Argentina's ecoregions.

## Author Contributions

**Arian Avalos:** conceptualization (equal), data curation (equal), formal analysis (equal), methodology (equal), resources (equal), software (equal), validation (equal), writing – original draft (equal), writing – review and editing (equal). **Alejandra Scannapieco:** data curation (equal), methodology (equal), validation (equal), visualization (equal), writing – original draft (equal), writing – review and editing (equal). **A. Carolina Monmany‐Garzia:** methodology (equal), validation (equal), visualization (equal), writing – original draft (equal), writing – review and editing (equal). **Ravi Kiran Donthu:** conceptualization (equal), data curation (equal), formal analysis (equal), investigation (equal), methodology (equal), software (equal), supervision (equal), validation (equal). **José Marcelino:** conceptualization (equal), data curation (equal), formal analysis (equal), investigation (equal), visualization (equal), writing – review and editing (equal). **Rosanna Giordano:** conceptualization (equal), data curation (equal), formal analysis (equal), funding acquisition (equal), investigation (equal), methodology (equal), project administration (equal), resources (equal), validation (equal), writing – review and editing (equal). **Tugrul Giray:** conceptualization (equal), data curation (equal), formal analysis (equal), funding acquisition (equal), investigation (equal), methodology (equal), project administration (equal), resources (equal), supervision (equal), writing – original draft (equal), writing – review and editing (equal). **Alberto Galindo‐Cardona:** data curation (equal), formal analysis (equal), investigation (equal), methodology (equal), visualization (equal), writing – original draft (equal), writing – review and editing (equal).

## Conflicts of Interest

The authors declare no conflicts of interest.

## Supporting information


**Data S1:** ece372233‐sup‐0001‐Supinfo01.xlsx.

## Data Availability

All the required data are uploaded as Supporting Information. Genotype data used in this study is publicly available through the source study: Donthu et al. (2024).
